# Developmental and epileptic encephalopathy in patients with epilepsy due to hypothalamic hamartomas

**DOI:** 10.1111/epi.18404

**Published:** 2025-04-10

**Authors:** Kathrin Wagner, Theo Demerath, Sarah Metzger, Friederike Niedermoser, Birgitta Metternich, Lisa Putzar, Horst Urbach, Victoria San Antonio‐Arce, Kerstin Alexandra Klotz, Andreas Schulze‐Bonhage

**Affiliations:** ^1^ Department of Neurosurgery, Epilepsy Center Medical Center – University of Freiburg Freiburg Germany; ^2^ Department of Neuroradiology Medical Center – University of Freiburg Freiburg Germany; ^3^ Department of Neuropediatric and Muscle Disorders Medical Center – University of Freiburg Freiburg Germany

**Keywords:** behavior, developmental and epileptic encephalopathy, intelligence

## Abstract

**Objective:**

What factors influence cognition and behavior in patients with epilepsy caused by hypothalamic hamartoma (HH)?

**Methods:**

We conducted a retrospective study of 103 patients referred to the Epilepsy Center in Freiburg, Germany, over the past 24 years. Analyzed parameters included development/intellectual functioning, behavior, seizure types and frequency, as well as electroencephalography (EEG) and magnetic resonance imaging (MRI) analyses.

**Results:**

Half of the patients showed signs of global developmental delay (GDD) or intellectual disability (ID). Patients with GDD/ID were younger at epilepsy onset (*p* < .05) and at first referral (*p* < .001), had shorter disease durations (*p* < .01), experienced more frequent seizures (*p* < .001), and were prescribed more antiseizure medication (ASM; *p* < .01). They also had larger HH volumes (hamartoma types Delalande III and IV, both *p* < .001) and more frequent pathological EEG background activity (*p* < .001), as well as more extended interictal epileptiform discharges (IEDs; *p* < .05, the rate of IED and seizure types were comparable, *p* > .05). Of interest, pathological EEG background activity and HH type were the only predictors of GDD/ID resulting in a highly predictive model (*R* ^2^ = 0.75, *p* < .001). Patients with GDD/ID also experienced more externalized behavioral problems, particularly aggression, which was predicted only by EEG background activity (*R* ^2^ = 0.36, *p* < .001). None of the epilepsy‐specific parameters, such as duration and seizure type or frequency, were significant predictors.

**Significance:**

Our findings support the idea that patients with epilepsy due to HH and GDD/ID may have a more severe underlying condition with a likely genetic etiology, characterized by developmental and epileptic encephalopathy.

**Key points**

- Patients with epilepsy due to hypothalamic hamartoma (HH) and global developmental delay/intellectual disability (GDD/ID) appear to have a more severe underlying condition, likely genetic in nature.
- Our results support the assumption of developmental and epileptic encephalopathy in these patients.
- These findings indicate the need for a thorough assessment of these patients, which could lead to improved individualized counseling and treatment.

## 1 INTRODUCTION

Hypothalamic hamartomas (HHs) are rare, non‐progressive malformations that occur during fetal development. They can become symptomatic through epileptic seizures, hormonal changes causing central precocious puberty (CPP), developmental delay, and behavioral abnormalities. The cognitive phenotype of these patients is very diverse, ranging from severely intellectually impaired patients to patients with average intellectual functioning (AIF).^1^ Furthermore, behavioral problems are very common in children with epilepsy due to HH,^2^ especially externalized behavior and diagnoses such as oppositional defiant disorder, aggression, and attention‐deficit/hyperactivity disorder (ADHD).

Various factors have been discussed that influence intellectual functioning and behavior. Deonna and Ziegler introduced a model of “an epileptic” developmental disorder,^3^ that states, uncontrolled epilepsy is the cause of global developmental delay (GDD) in children and intellectual disability (ID) in adults. Although seizure frequency and severity correlate with cognitive impairment,^4^ there are reports of children with developmental delay before the onset of seizures.^5^, ^6^ In addition, some studies did not find an association between age at epilepsy onset or disease duration and GDD/ID.^6^, ^7^ On the other hand, the size of the hamartoma^7^, ^8^, ^9^ appears to be associated with cognitive and behavioral disturbances, whereas the role of CPP remains not fully understood. On the one hand, a correlation between CPP and GDD/ID was found,^1^ but, of interest, patients who were referred to an endocrinology outpatient clinic because of CPP due to HH showed mostly average cognitive performance. Only one third of them had seizures and only some of them revealed borderline intellectual functioning.^10^

In this cross‐sectional study, we analyzed data of all patients with epilepsy due to HH who were referred to the epilepsy center in Freiburg, Germany, over more than 20 years. The aim of this study was to examine differences between patients with AIF compared to those with GDD/ID and identify potential influencing factors on cognition and behavior. To our knowledge, no study to date has analyzed such a large cohort of patients with HH, including their electroencephalography (EEG) abnormalities, hamartoma volumes, and other clinical features in relation to intellectual functioning and behavior.

## 2 MATERIALS AND METHODS

We retrospectively studied patients with epilepsy due to a HH who were examined at the Epilepsy Center in Freiburg, Germany, between 1999 and 2023. Their medical files and neuropsychological, EEG, and MRI examinations were assessed to analyze their cognitive abilities, behavior, seizures, EEG features, and MRI results. The study was approved by the local ethics committee.

### 2.1 Cognitive assessment

According to the Diagnostic and Statistical Manual of Mental Disorders, Fifth Edition (DSM‐5), intellectual disability (or ID) involves impairments of general mental abilities that impact adaptive functioning in three domains starting in the developmental period:

1. The *conceptual domain* includes skills in language, reading, writing, math, reasoning, knowledge, and memory.
2. The *social domain* refers to empathy, social judgment, interpersonal communication skills, the ability to make and retain friendships, and similar capacities.
3. The *practical domain* centers on self‐management in areas such as personal care, job responsibilities, money management, recreation, and organizing school and work tasks.

In children younger than 5 years of age, GDD describes an important developmental milestone delay concerning motor, language, cognition, social functioning, and activities of daily living.

For every patient, it was determined if an ID or GDD was present at the time of referral according to impairments in the domains described. Information was taken from present neuropsychological and clinical assessments, including information based on parents' reports as well as previous medical reports. Only if clearly reported or assessed impairments in all three domains were found and were already reported during the developmental period was an ID/GDD indicated. Patients without formal intelligence quotient (IQ) assessment but with average education and work within the regular labor market, social integration, and an independent living situation were classified as AIF. Two independent raters (K.W. and B.M.) classified the patient sample.

### 2.2 Behavioral assessment

Information on behavior was assessed from present neuropsychological and clinical examinations, including information from parents as well as previous medical reports. If behavioral problems were found, they were categorized as internalized (depressive, anxious, social withdrawal, somatic complaints, etc.), externalized (aggressive behavior, ADHD, tantrums, etc.), or both. Furthermore, the occurrence of any aggressive behavior (against oneself, others, or objects) was registered separately. Two independent raters (K.W. and V.S.A.‐A.) classified the patient sample.

### 2.3 Seizure types

Seizure types were determined by categorizing every seizure‐reported semiology until the time of the first referral: focal emotional (gelastic), all other focal (non‐gelastic), and focal to bilateral tonic–clonic seizures. For each patient, the most severe seizure type was determined. Gelastic seizures are focal seizures that may sound like laughter or look like a smile, but individuals generally do not experience happy feelings. They can be associated with little or no change in consciousness and are very common in patients with HH. They were rated as the least disabling seizure type. Focal non‐gelastic seizures refer to all other seizures with focal onset. As they occur more often with impaired awareness, they were classified as more severe than purely gelastic seizures. (Focal to) bilateral tonic–clonic seizures were rated as the most disabling seizures.

### 2.4 Seizure frequency

Seizure frequency was defined as reported by patients at their first referral combined for all seizure types: more than once per day, one seizure per day, at least once per week, at least once per month, less than once per month.

### 2.5 EEG analyses

For the rating of background activity, we analyzed a 20 min section in the awake state. For estimation of the spike count, two 5‐min sections during sleep stages II to III (patients with video‐EEG [vEEG] only) with minimal artifacts were selected; a 2‐h interval was maintained from the preceding and subsequent seizure events whenever possible. EEG was visually analyzed by two independent raters (S.M., F.N., or K.A.K.). All disagreements were resolved after a consensus reading.

EEG background activity was scored as normal if all of the following criteria were met (see Table S1, ^11^, ^12^): (1) frequency of background activity within the normal age range (see below); (2) dominance over the posterior brain areas; (3) normal reactivity to (active or passive) eye opening.

Interictal epileptiform discharges (IEDs) were identified based on the current International Federation of Clinical Neurophysiology (IFCN) criteria^13^ and the rate (per second) was calculated. Distinct foci of one patient were identified based on their localization and morphology. The extent of these typical foci were assessed and all foci of one patient were summarized and categorized into the following categories: one lobe, two lobes unilaterally, more than two lobes unilaterally, bilaterally independent, bilaterally synchronous.

### 2.6 MRI analyses

MRI scans were retrospectively available in 103 patients (55 AIF, 48 GDD/ID) and analyzed by a board‐certified neuroradiologist with 8 years of experience in clinical neuroimaging (T.D.) to classify the individual type of HH according to Delalande,^14^ and to determine the attachment side of the HH (unilateral right/left or bilateral). In addition, HHs were manually segmented on isotropic three‐dimensional (3D) T1‐weighted (T1w) images (resolution 1 mm^3^) based on hypointense T1 signal properties using our in‐house post‐processing platform NORA (www.nora‐imaging.org) and individual hamartoma volumes were extracted.

### 2.7 Statistical analyses

In order to examine differences between patients with AIF compared to those with GDD/ID and identify potential influencing factors on cognition and behavior, demographic and clinical findings were compared between patients with and without GDD/ID. Continuous variables (age, age at epilepsy onset, disease duration, IQ, number of antiseizure medications [ASMs], IEDs, and HH volume) were analyzed using *T* tests. Nominal or ordinal variables were compared with chi‐square tests or Mann–Whitney *U* tests (gender, behavioral problems, most severe seizure type, pathological EEG activity, HH attachment, HH type, seizure frequency, CPP).

Age at referral, age at epilepsy onset, disease duration, seizure frequency, EEG background activity, number of ASMs, HH volume, HH type, and CPP were evaluated in a binary logistic regression analysis as influencing factors, with GDD/ID defined as the dependent variable (Method: enter). In further binary logistic regression analyses, internalized and externalized as well as overall behavioral problems served as the dependent variables.

## 3 RESULTS

Between 1999 and 2023, a total of 111 patients with epilepsy caused by a HH were referred to the Epilepsy Center, Freiburg. For all patients, it could be determined whether a GDD/ID was present or not. A second rater independently classified the patient sample with a concordance of 95.5%. Disagreements were resolved after a consensus reading. EEG data were available retrospectively for 110 patients. In 80 of these patients, vEEG registrations were recorded during presurgical workup, but only 63 patients had data available for evaluation of background activity and IEDs. Routine EEG was carried out for the remaining 30 patients to assess background activity. MRI scans were available for 103 patients (see Figure S1 for data flow overview).

Of the final sample of 103 patients, 23 had undergone at least one surgical intervention previously (see Table 1), which did not result in seizure freedom. For an overview, see Table 1.

**TABLE 1:** Demographical and clinical data of the final patient sample of 103 patients.

	Range/frequency	Mean ± SD
Age	1–59 years	20.1 ± 14.7 years
Age at epilepsy onset	0–55 years	3.3 ± 6.4 years
Disease duration	0–55 years	16.5 ± 13.0 years
Gender	48/55 female/male	
Treatment beforehand (3 patients received 2 different treatments)	80 ASM only 8 VNS (deactivated in 2) 8 Radiosurgery 7 Partial resection 3 Endoscopic partial disconnection	

Abbreviations: ASM, antiseizure medication; SD, standard deviation; VNS, vagus nerve stimulation.

According to the DSM‐5 criteria, almost half of the patients (*N* = 48, 47%) showed GDD or ID, respectively, whereas 55 patients (53%) had borderline or average intellectual functioning. A comparison of these clinically diverse patient groups showed that patients with GDD/ID were significantly younger at epilepsy onset (*p* < .05) as well as at first referral (*p* < .001), and had shorter disease durations (*p* < .01). The frequency of prior surgeries was similar in both groups (*p* > .1). The majority of both groups were treated with ASMs only at the time of assessment (83.6% and 70.8%).

**FIGURE 1:**
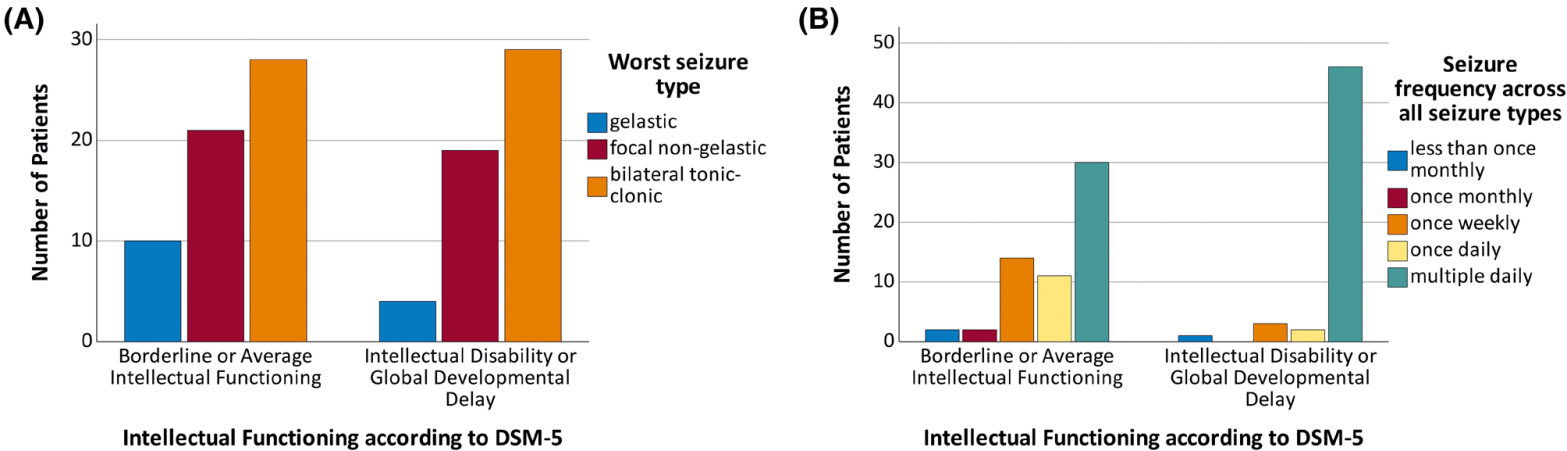
Most severe seizure type reported was comparable between patients groups (left, *p* > .1). Seizure frequency reported was higher in patients with global developmental delay or intellectual disability (right, *p* < .01).

Of interest, seizure types until referral were comparable between groups (*p* > .1; see Figure 1, left), whereas in patients with GDD/ID multiple seizures per day (*p* < .01, see Figure 1 right) as well as a larger number of ASM were significantly more common (*p* < .01).

**FIGURE 2:**
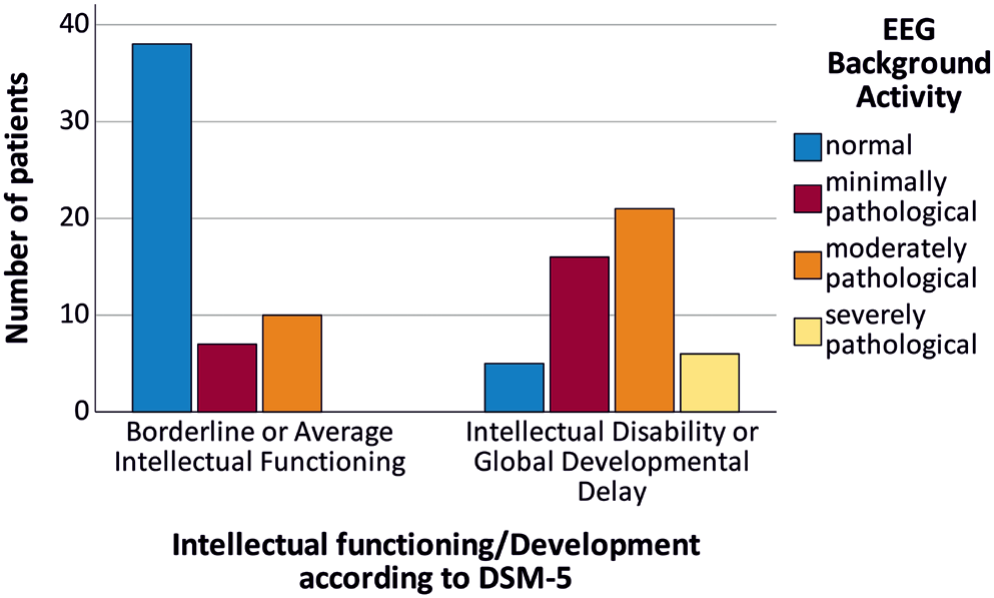
Patients with global developmental delay or intellectual disability had significantly more often moderately or severely pathological EEG background activity (*p* < .001).

Furthermore, patients with GDD/ID also had significantly more frequent pathological EEG background activity (*p* < .001, see Figure 2). In the subgroup with available IED data (*N* = 63, 27 with GDD/ID, 36 AIF), cognitively impaired patients also exhibited a greater extent of IEDs (*p* < .05, see Table 2), whereas the rate of IEDs was similar between groups (*p* > .05).

**TABLE 2:** Comparison of the two patient groups.

	Patients with intellectual disability (*N* = 48)	Patients without intellectual disability (*N* = 55)	*p*‐Value
Age at referral, mean ± SD	15.1 ± 11.1 years	24.4 ± 16.0 years	*p* < .001*
Age at epilepsy onset, mean ± SD	1.9 ± 2.7 years	4.5 ± 8.2 years	*p* < .05*
Disease duration, mean ± SD	13.0 ± 10.4 years	19.6 ± 14.3 years	*p* < .01*
Gender, female/male	21/27	27/28	n.s.^#^
IQ/DQ, mean ± SD^$^	63.2 ± 12.6 (*N* = 26)	93.8 ± 13.3 (*N* = 29)	*p* < .001*
Behavioral problems			
Internalized	15	27	*p* = .05^#^
Externalized/aggression	33/25	20/8	*p* < .001^#^
Most severe seizure type			
focal gelastic/focal non‐gelastic/bilateral tonic–clonic	4/18/26	9/21/25	n.s.^#^
Seizure frequency	Most frequent category:	Most frequent category:	
Across all seizure types	89.6% multiple daily	54.5% multiple daily	*p* < .001^§^
Only bilateral tonic–clonic	27.1% less than 1/month	25.5% less than 1/month	n.s.^§^
Number of ASMs, mean ± SD/median	2.3 ± 0.9/2	1.8 ± 0.8/2	*p* < .01*
EEG background activity normal/minimally path./moderately path./severely path.	5/16/21/6	38/7/10/0	*p* < .001^#^
Interictal epileptiform discharges			
Rate, per s	2.4 ± 10.7 (*N* = 26)	3.2 ± 16.9 (*N* = 39)	n.s.*
Extent, 1 lobe/2 lobes unilateral/>2 lobes unilateral/bilateral independent/bilateral synchronous	2/0/1/11/13 (*N* = 27)	9/3/1/18/5 (*N* = 39)	*p* < .05^#^
Hamartoma volume, in ml	2.8 ± 3.6 (*N* = 50)	0.8 ± 1.2 (*N* = 53)	*p* < .001*
Hamartoma attachment, unilateral/bilateral	25/23	37/18	n.s.^#^
Hamartoma type, Delalande I/II/III/IV	Median = 3 2/12/17/17	Median = 2 13/30/10/2	*p* < .001^#^
Central precocious puberty, yes/no/unknown	23/22/3	8/43/4	*p* < .01^#^

*Abbreviations*: ASM, antiseizure medication; DQ, developmental quotient; IQ, intelligence quotient; ml, milliliter; path., pathological; s, second; SD, standard deviation. *T test, ^#^Chi‐squared test, ^§^Mann–Whitney *U* test, n.s., not significant (*p* > .05), ^$^age‐appropriate Wechsler Intelligence Test (*N* = 43), Bayley Scales of Infant and Toddler Development (*N* = 9), Kaufman Assessment Battery for Children (*N* = 3).

**FIGURE 3:**
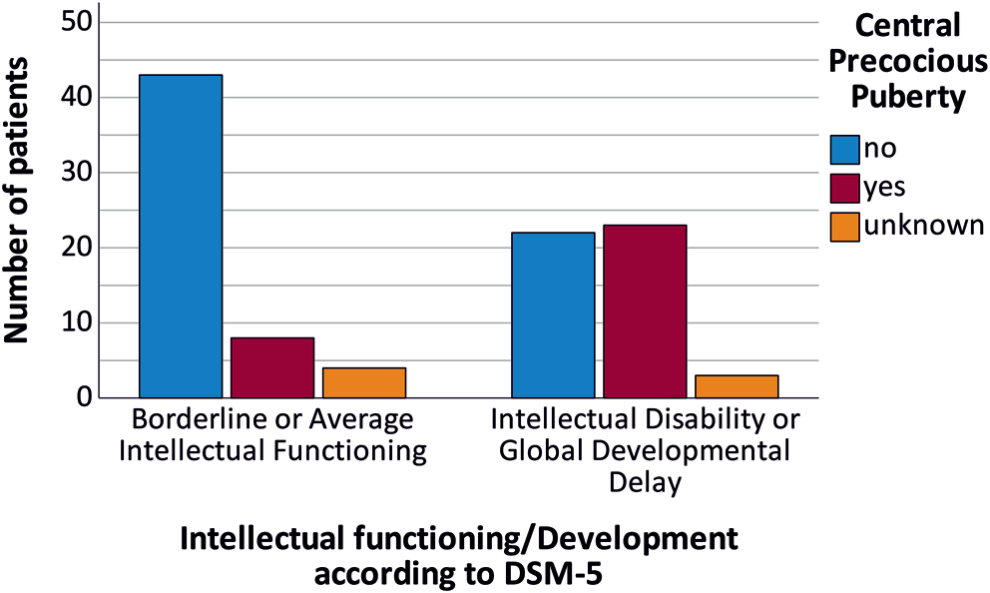
In about half of the patients with global developmental delay or intellectual disability (51%) central precocious puberty was present, whereas it was diagnosed in only 16% of patients with average development or intellectual functioning (*p* < .01).

MRI analyses revealed that HHs of cognitively impaired patients were significantly larger (*p* < 0.001, see Table 2) and thus more often classified as Delalande types III and IV (*p* < .001). There was no group difference in the type of attachment (uni‐ or bilateral) of the HHs (*p* > .05). In addition, central precocious puberty (or CPP) was more common in patients with GDD/ID than in patients with AIF (*p* < .01, see Figure 3). Further examination revealed that about half of the patients with GDD/ID had CPP. Of interest, patients with GDD/ID *and* CPP had significantly larger hamartomas than patients with AIF (*p* < .05). For a comprehensive overview refer to Table 2.

Valid information on development before the onset of epilepsy was available in 65 patients (63%). In five patients, no retrospective information was found. Thirty‐three patients had their first seizure during the first year of life, making it difficult to retrospectively evaluate their development before disease onset. Of the 65 patients with available information, 49 (75%) had developed normally, whereas only 16 patients (25%) had developmental delay and/or behavioral problems reported before epilepsy onset. Patients with developmental delay and/or behavioral problems before epilepsy onset had larger HH volumes (*p* < .01), a younger age at epilepsy onset, and shorter disease duration (both *p* < .05) compared to patients who had shown normal development before the first seizure was reported (Mann–Whitney *U* test).

**FIGURE 4:**
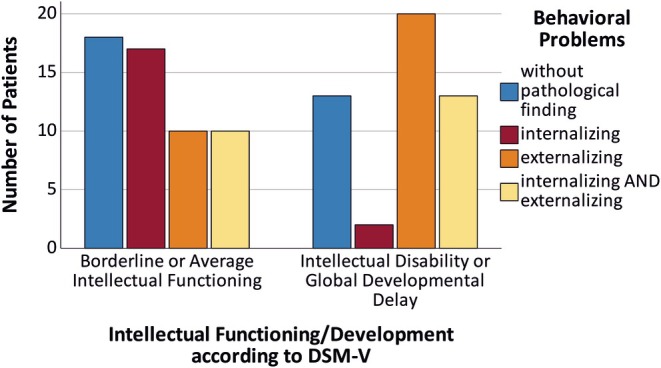
Patients with global developmental delay or intellectual disability exhibited significantly more often externalized and less internalized behavioral problems than patients with average development or intellectual functioning (*p* < .05).

Behavioral problems were found in 69.9% of the patients and were more often externalized (and concomitant with aggression) in patients with GDD/ID (both *p* < .001) and more often internalized in patients without GDD/ID (*p* < .05, see Figure 4). Regarding specific neurodevelopmental conditions, like autism and ADHD, very few patients had received a full diagnostic assessment, even though ADHD‐ and autism‐like behavior were reported. Sufficient information was available for only 21 patients with ADHD‐like symptoms and 23 patients with autism‐like behavior, respectively, and led to a diagnosis of ADHD in 4 patients (3 of them with GDD/ID, Pearson chi‐square *p* < .01) and autism in 4 patients (all with GDD/ID, Pearson chi‐square *p* < .01).

Significant predictors of GDD/ID were pathological EEG background activity (odds ratio [OR] = 8.41, 95% confidence interval [CI]: 2.69–26.30, *p* < .001) and a larger, more attached hamartoma type (OR = 8.87, 95% CI: 2.2–35.80, *p* < .01). Age, age at onset, disease duration, seizure frequency, ASM load, HH volume, and CPP (all *p* > .1) did not explain additional variance and therefore were not significant predictors. The model had a very good predictive value: It explained 79% of the variance (Nagelkerkes *R* ^2^ = 0.75, *p* < .001) and 82.5% of the patients were classified correctly.

No significant variables could be identified to predict general behavioral or internalized problems (both models *p* > .1). However, the number of ASMs (OR = 1.72, 95% CI: 0.97–3.05, *p* = .06) and the HH volume (OR = 0.81, 95% CI 0.64–1.03, *p* = .08) showed trends in predicting externalized behavioral problems (Nagelkerkes *R* ^2^ = 0.33, *p* < .001, 72.8% correct classifications). Aggressive behavior could be predicted by EEG background activity (OR = 1.83, 95% CI: 1.04–3.23, *p* < .05; Nagelkerkes *R* ^2^ = 0.36, model *p* < .001, 77.7% correct classifications), whereas none of the other factors reached significance (*p* > .05).

## 4 DISCUSSION

To our knowledge, this is the largest retrospective cross‐sectional study of patients with epilepsy due to HH that investigates various factors influencing cognitive development. In our patient group, cognitive development was most accurately predicted by EEG background activity and HH type. Patients with GDD/ID more frequently exhibited pathological EEG background activity, and in a subgroup, we also observed more bilateral synchronous IEDs compared to patients with AIF. Of interest, their rate of epileptic discharges was comparable. Pathological EEG background activity is a known sign of brain dysfunction or even encephalopathy, and can be seen in various neurological disorders (e.g., subcortical lesions, see Ref.^15^) and developmental disorders (e.g., autism, see Ref.^16^). Marked background pathology may disrupt normal cortical processes like learning, thus having a profound negative impact on development.^17^

Specific epilepsy features such as IED rate and seizure frequency did not predict GDD/ID. Furthermore, the duration, since diagnosis of epilepsy was even shorter in these patients. Therefore, the period in which epileptic activity and seizures could have negatively influenced development cannot explain the developmental delay in our cohort. The clinical presentation of the impaired group with an earlier onset, larger hamartomas, and more frequent seizures, suggests that they had a more severe underlying disease. This may be the common underlying reason for developmental delay (in some cases even before the first seizure was recognized), for larger and more difficult to treat hamartoma types, for more frequent seizures (associated with more ASMs), and for pathological EEG background activity. Some of these aspects may have intensified the developmental delay over time. For instance, epilepsy onset at a younger age may have disrupted sensitive and critical periods of early development leading to delay.

In about 63% of patients, information on development before epilepsy onset was available. Of interest, the small subgroup of patients who already had GDD/ID before seizures started showed larger hamartomas, younger age at onset, and shorter disease durations at the time of evaluation. Other clinical features were comparable to patients without developmental delay before epilepsy onset. This may also be a sign of a more severe underlying primary disease that led to earlier and stronger symptoms. Beyond that, a natural history study would be beneficial to understand the disease course and its influences on development and behavior. However, data collection over time without therapy is prohibited due to ethical concerns. Previous studies^6^, ^18^ have described GDD independent of seizures, supporting the notion that the HH itself and/or its underlying etiology disrupts normal development. As an underlying etiology, genetic mutations, like somatic mutations in sonic hedgehog pathway genes have been discussed.^19^ For other epilepsies with a marked phenotypic heterogeneity, for example, *SCN1A*‐related epilepsies, the relevance of additional genomic variation (beyond *SCN1A*) has been shown.^20^ The influence of the “genomic background” on disease severity should be explored further for patients with HH as well.

Moreover, the presence of additional neurodevelopmental conditions in addition to having seizures would be a sign of a more severe underlying disease. Another study^6^ described autistic behavior in children with HH before the onset of seizures, particularly generalized seizures or even in the absence of any seizures. These findings support the assumption that autistic features are more likely attributable to the HH itself and/or the underlying etiology, likely genetic, than directly to seizures or an epileptic encephalopathy.^6^ In our cohort, only 20% to 22% of the patients had sufficient information on additional neurodevelopmental conditions (ADHD and autism spectrum disorder [ADS], respectively) available. Hence, formal diagnoses of ADHD and autism were scarce, however these conditions were almost exclusively diagnosed in patients with GDD/ID, supporting the notion of a more severe underlying disease.

Larger HH volumes (and therefore more often HH Delalande type IV) were seen in patients with GDD/ID, and the type of HH was predictive of cognitive impairment. Previous studies have also found an association between larger HHs and more severe cognitive impairment.^1^, ^8^, ^9^ The hypothalamus is known to be an integrative structure with many afferent and efferent connections comprising a variety of different functions, such as vital bodily processes including cardiovascular regulation, sleep, metabolism, stress, thermoregulation, water and electrolyte balance, appetite regulation, sexual behavior, and endocrine and immune responses.^21^ Via its connections to the mammillary bodies, the thalamus, the hippocampus, the amygdala, and various cortical regions like the frontal lobe, it integrates functions such as learning and memory, alertness, and emotional regulation.^22^ Therefore, its disturbance might affect cognitive and behavioral development. Any interference of hypothalamic function can have a negative effect on intellectual capabilities, irrespective of the seizure control.^1^ In patients with craniopharyngiomas primarily affecting the hypothalamus, a wide range of hypothalamic dysfunctions can also be observed: a complex set of cognitive symptoms (dementia‐like, Korsakoff‐like) as well as emotional (aggressiveness, depression), and behavioral (autism‐like) disturbances have been described.^23^, ^24^

A larger HH size also correlated with CPP.^1^, ^25^ However, occurrence of CPP alone could not explain GDD/ID, as illustrated in Figure 4 on the right side. Only half of the patients with GDD/ID had CPP. Patients with larger HH volumes *in addition to* CPP were more likely to exhibit GDD/ID. Thus, CPP alone does not seem to fully explain the presentation of GDD/ID, indicating that additional factors such as HH volume or type need to be considered.

Many patients presented behavioral problems that were more often externalized (accompanied by aggression) in cognitively impaired patients, and more often internalized in patients without GDD/ID. These results support previous findings on behavioral problems, particularly aggression in patients with epilepsy due to HH.^26^, ^27^ In our patients, abnormal EEG background activity was the only factor that could predict aggressive behavior. A systematic review identified male gender, younger age at the time of the first seizure, presence of ID, and multiple seizure types as predictors of aggression in patients with HH, whereas seizure frequency and a history of CPP were not.^28^ None of the studies analyzed the association between EEG features and aggression. However, the hypothalamus has been shown to be part of neural circuits related to aggression that also regulate other aspects of social behavior.^29^ HH volume and ASM load were by trend predictors for externalized behavioral problems. Larger HH volumes impair larger areas and are more likely to disturb the function of the hypothalamic nuclei responsible for the regulation of stress control and aggression.^30^ Internalized problems like depression and anxiety were rarely described in patients with GDD/ID, but can easily be overlooked in these patients.

There are some limitations to our study. Data were collected retrospectively, so not all aspects were available for the evaluated time span of 24 years. There may have been a bias toward more severe cases, as these patients are often referred to specialized epilepsy centers. In addition, standardized assessment in severely impaired patients was very limited. Information was gathered on assistance needed for everyday activities (including kindergarten, school, or work) as well as social integration and behavior from our own and prior reports. Presence and frequency of gelastic seizures were possibly underreported, whereas records of bilateral tonic–clonic seizures were likely the most reliable. Gelastic seizures can often be missed in children, leading to a later age at diagnosis and estimated onset of epilepsy. Some other studies have found an earlier age at onset of seizures compared to our group without intellectual disabilities. For example Nguyen et al.^5^ reported a mean age of 2.5 years and a median of 1 year, whereas our unimpaired group had a mean age at onset of 4.5 (SD = 8.2) and a median of 2 years. The unimpaired group included one outlier who was referred to us at the age of 55 years when focal seizures with impaired awareness occurred in addition to focal aware gelastic seizures (age at onset was 4). The age at onset reported in the literature is comparable to our patients with GDD/ID (mean age at onset 1.9, median of 1 year).

Some patients had series of seizures (e.g., series of tonic seizures), which were not accounted for with the chosen seizure frequency categories. Retrospectively, seizure frequencies for the different seizure types could not be reliably collected from the medical records. Consequently, we reported seizure frequencies combined for all seizure types.

## 5 CONCLUSION

This study describes the largest cohort of patients with epilepsy due to HHs and a wide range of developmental and behavioral profiles to date. Our findings support the notion that patients with GDD/ID have a more severe underlying disease, associated with a younger age at epilepsy onset, pathological EEG background activity, larger HH volumes, higher seizure frequencies, externalized behavioral problems such as aggression, and intake of more ASMs. In addition, specific epilepsy attributes such as higher seizure frequencies and more extended IEDs may have intensified their cognitive and behavioral problems. Overall, we propose to consider HHs a developmental as well as an epileptic encephalopathy, rather than solely an epileptic encephalopathy. Further investigation into the underlying genetic etiology is needed to fully understand the diverse clinical phenotype of this syndrome.

## AUTHOR CONTRIBUTIONS

K.W.: Substantial contributions to the conception and design of the work, acquisition, analysis, and interpretation of data for the work; drafting the work and revising it critically for important intellectual content; final approval of the version to be published; and agreement to be accountable for all aspects of the work in ensuring that questions related to the accuracy or integrity of any part of the work are appropriately investigated and resolved. T.D.: Substantial contributions to acquisition, analysis, and interpretation of data for the work; drafting the work and revising it critically for important intellectual content; final approval of the version to be published; and agreement to be accountable for all aspects of the work in ensuring that questions related to the accuracy or integrity of any part of the work are appropriately investigated and resolved. S.M.: Substantial contributions to acquisition, analysis, and interpretation of data for the work; revising the work critically for important intellectual content; final approval of the version to be published; and agreement to be accountable for all aspects of the work in ensuring that questions related to the accuracy or integrity of any part of the work are appropriately investigated and resolved. F.N.: Substantial contributions to acquisition, analysis, and interpretation of data for the work; revising the work critically for important intellectual content; final approval of the version to be published; and agreement to be accountable for all aspects of the work in ensuring that questions related to the accuracy or integrity of any part of the work are appropriately investigated and resolved. B.M.: Substantial contributions to acquisition and interpretation of data for the work; revising the work critically for important intellectual content; final approval of the version to be published; and agreement to be accountable for all aspects of the work in ensuring that questions related to the accuracy or integrity of any part of the work are appropriately investigated and resolved. L.P.: Substantial contributions to interpretation of data for the work; revising the work critically for important intellectual content; final approval of the version to be published; and agreement to be accountable for all aspects of the work in ensuring that questions related to the accuracy or integrity of any part of the work are appropriately investigated and resolved. H.U.: Substantial contributions to interpretation of data for the work; revising the work critically for important intellectual content; final approval of the version to be published; and agreement to be accountable for all aspects of the work in ensuring that questions related to the accuracy or integrity of any part of the work are appropriately investigated and resolved. V.S.A.: Substantial contributions to acquisition and interpretation of data for the work; revising the work critically for important intellectual content; final approval of the version to be published; and agreement to be accountable for all aspects of the work in ensuring that questions related to the accuracy or integrity of any part of the work are appropriately investigated and resolved. K.A.K.: Substantial contributions to acquisition, analysis, and interpretation of data for the work; revising the work critically for important intellectual content; final approval of the version to be published; and agreement to be accountable for all aspects of the work in ensuring that questions related to the accuracy or integrity of any part of the work are appropriately investigated and resolved. A.S.B.: Substantial contributions to interpretation of data for the work; revising the work critically for important intellectual content; final approval of the version to be published; and agreement to be accountable for all aspects of the work in ensuring that questions related to the accuracy or integrity of any part of the work are appropriately investigated and resolved.

## FUNDING INFORMATION

None

## CONFLICT OF INTEREST STATEMENT

None of the authors has any conflict of interest to disclose.

## ETHICS STATEMENT

We confirm that we have read the Journal's position on issues involved in ethical publication and affirm that this report is consistent with those guidelines. The study was approved by the local ethics committee.

## CONSENT

Written patient consent was not applicable due to the study design of retrospective clinical data analysis.

## CLINICAL TRIALS REGISTERATION

The study was registered at the German Clinical Trials Register.

## Supporting information


Figure S1.

Table S1.

## Data Availability

The data that support the findings of this study are available from the corresponding author upon reasonable request.
