# Supplementary material for: Developmental and epileptic encephalopathy in patients with epilepsy due to hypothalamic hamartomas
Source: Epilepsia. 2025 Apr 10;66(8):2894–903. doi: 10.1111/epi.18404 (PMC12371641; doi:10.1111/epi.18404)
Supplement: Supplementary file 2 — Table S1. [file EPI-66-2894-s001.docx]

**Supporting table 1: Scoring criteria for evaluation of EEG background activity.**

| **Scoring background activity** | |  |  |  |  |
| --- | --- | --- | --- | --- | --- |
|  |  | **Frequency background activity* (s.below )** | **Embedded slow waves** | **Structure** | **Reagibility** |
| I | normal | within normal range | no | normal | normal |
| II | minimally pathological | within normal range | yes | reduced | reduced |
| III | moderately pathological | below normal range | not applicable | reduced | reduced |
| IV | severely pathological | not detectable/ encephalopathic | not applicable | not applicable | not ratable |

*Frequency background activity rated as pathological: age <12 months: <5 Hz, 13-47 months: <6Hz, 48-71 months: <7 Hz, 72-95 months: <8hz, ≥96 months: ≤8 Hz (11, 12).
